# Propyl gallate inhibits TPA-induced inflammation via the nuclear factor-κB pathway in human THP-1 monocytes

**DOI:** 10.3892/etm.2013.896

**Published:** 2013-01-16

**Authors:** HUNG-CHIH HSU, WAN-CHEN LIN, PEY-JIUM CHANG, CHANG-ZERN HONG, CHING-HSEIN CHEN

**Affiliations:** 1 Department of Physical Medicine and Rehabilitation, Chang Gung Memorial Hospital, Chiayi;; 2 Graduate Institute of Clinical Medical Sciences, College of Medicine, Chang Gung University, Taoyuan;; 3 Department of Nursing, Chang Gung University of Science and Technology, Chiayi;; 4 Department of Microbiology, Immunology and Biopharmaceuticals, College of Life Sciences, National Chiayi University, Chiayi;; 5 Department of Physical Therapy, Hung Kuang University, Taichung, Taiwan, R.O.C.

**Keywords:** propyl gallate, anti-inflammation, cyclooxygenase-2, nuclear factor-κB

## Abstract

Propyl gallate (PG) is an antioxidant that has been used as an additive in several foods to protect against oxidation. The present study examined the anti-inflammatory effect of PG on 12-O-tetradecanoylphorbol-13-acetate (TPA)-induced inflammation in human THP-1 monocytes. Pretreatment with PG markedly inhibited the TPA-induced expression levels of cyclooxygenase-2 and prostaglandin E2. The application of PG significantly inhibited the nuclear translocation of p65, a subunit of nuclear factor-κB (NF-κB) and phosphorylation of p65 (Ser536) in TPA-treated THP-1 cells. PG also inhibited the phosphorylation of IκB and IκB kinase. These results indicate that PG inhibits the inflammatory response by blocking the NF-κB signaling pathway in TPA-induced THP-1 monocytes. Therefore, PG may be useful as a therapeutic agent in inflammatory diseases.

## Introduction

Propyl gallate (PG), an ester that is also known as propyl 3,4,5-trihydroxybenzoate and belongs to the polyphenolic compound family, is synthesized by the condensation of propanol and gallic acid. PG is used in foods, cosmetics, hair products, adhesives and lubricants due to its antioxidative properties, which protect foods containing oils and fats from oxidation by hydrogen peroxide and free oxygen radicals.

The formation, metabolism, catabolism, and physiological and pathophysiological roles of prostanoids are associated with the development of inflammation and carcinogenesis (1). Cyclooxygenase-2 (COX-2) is an enzyme that is critical for prostanoid synthesis (2). This process is initiated by the enzymatic release of arachidonic acid (AA) from cellular stores by phospholipase A2 (3). AA is then metabolized to prostaglandins via the COX-2 pathway through a series of enzymatic steps (4). COX-2 is inducible and may be associated with one or more pathophysiological states or reactions (5). Since a number of the metabolic steps in this pathway generate reactive oxygen species (6), it is important to develop COX-2-specific agents that are effective anti-inflammatory drugs or have cancer preventive activity.

The discovery of the molecular associations between inflammation and cancer was a major breakthrough in chemoprevention research. Components of the cell signaling network have been implicated in the promotional stage of carcinogenesis, particularly those that converge on the redox-sensitive transcription factor nuclear factor-κB (NF-κB), which is involved in mediating the inflammatory response (7,8). One of the major target molecules subjected to NF-κB-driven transactivation is COX-2, which is involved in the biosynthesis of prostaglandins and inflammation (9). Inappropriate upregulation of COX-2 has frequently been observed in various premalignant and malignant tissues (10). The role of abnormally high levels of COX-2 in tumorigenesis has further been corroborated by the increased susceptibility of COX-2-overexpressing mice (11) and the relatively increased resistance of COX-2-knockout animals to spontaneous or experimentally induced carcinogenesis (12). Therefore, targeted inhibition of COX-2 is regarded as a promising and practical approach to prevent cancer (13).

NF-κB generally exists as a heterodimer of the p50 and RelA (p65) polypeptides, bound in an inactive state in the cytoplasm by the inhibitor protein IκB (14). Following cellular stimulation by a variety of agents, IκB is phosphorylated and degraded by the proteasome, allowing NF-κB to translocate to the nucleus and regulate the expression of the target genes, including a number that control cellular growth properties and apoptotic cell death (15,16). The transactivation of NF-κB-regulated genes requires not only the binding of NF-κB to the promoter regions, but also the phosphorylation of p65/RelA, which is the active subunit of NF-κB. Topical applications of 12-O-tetradecanoylphorbol-13-acetate (TPA) have been shown to cause an increase in p65/RelA phosphorylation at serine 536 (17).

PG is an antioxidant which affects and/or inhibits the inflammatory pathway. Although it appears that the inhibition of TPA-induced COX-2 by PG occurs through the NF-κB pathway, the details of the mechanism remain to be elucidated. In the present study, TPA-induced COX-2 was used in human THP-1 cells as an inflammation model to evaluate the effect of PG on COX-2 expression and prostaglandin E2 (PGE2) production. The effect of PG on the NF-κB pathway was also determined.

## Materials and methods

### Cell line and reagents

The human THP-1 cell line was obtained from the Bioresource Collection and Research Center (Hsinchu, Taiwan, R.O.C.). Roswell Park Memorial Institute-1640 (RPMI-1640) and fetal bovine serum (FBS) were obtained from Hyclone (South Logan, UT, USA). Anti-COX-2, anti-NF-κB, anti-IκB, anti-IKKα and anti-β-actin primary antibodies and a secondary antibody labeled with horseradish-peroxidase were purchased from Santa Cruz Lab Vision (Santa Cruz, CA, USA). Anti-phospho-IKKα (Ser180)/IKKβ (Ser181), anti-p65/RelA and anti-phospho-p65 (Ser536) primary antibodies were purchased from Cell Signaling Technology, Inc. (Danvers, MA, USA). PG, TPA and other chemicals were purchased from Sigma Chemical Co. (St. Louis, MO, USA) and the PGE2 enzyme immunoassay kit was purchased from Stressgen (Ann Arbor, MI, USA).

### Cell culture and treatment

The basal medium for the THP-1 cell line culture was composed of RPMI-1640 supplemented with 10% FBS, 100 U/ml penicillin G and 100 *μ*g/ml streptomycin. The PG stock solution (100 mM) was dissolved in dimethylsulfoxide (DMSO) and various concentrations were prepared in the basal medium with a final DMSO concentration of 0.1%, which was considered to cause little or no damage in the THP-1 cells.

### PGE2 assay

A PGE2 EIA kit from Stressgen was used to measure the PGE2 secreted in conditioned cell culture media of serum-starved cells for 24 h with and without TPA or PG, according to the manufacturer’s instructions.

### Western blot analysis

Following treatment, cells were washed with phosphate-buffered saline (PBS), resuspended in a protein extraction buffer for 10 min, then centrifuged at 12,000 rpm for 10 min at 4°C to obtain the total extracted proteins. The protein concentrations were evaluated with a Bio-Rad protein assay reagent (Bio-Rad, Richmond, CA, USA). The expression levels of various intracellular proteins were then evaluated by western blot analyses. Briefly, the total extracted protein content was boiled in a loading buffer and an aliquot corresponding to 50 or 100 *μ*g protein was separated by 12% SDS-polyacrylamide gel. Following electrophoresis, proteins were electrotransferred onto a polyvinylidene fluoride transfer membrane. After blotting, the membranes were incubated with various primary antibodies overnight at 4°C and then washed with PBST solution (0.05% Tween-20 in PBS). Following washing, the secondary antibody, labeled with horseradish-peroxidase, was incubated for 1 h at room temperature and then washed with the PBST solution. The antigen-antibody complexes were detected by enhanced chemiluminescence using a chemiluminescence analyzer (Amersham Pharmacia Biotech, Piscataway, NJ, USA).

### Statistical analysis

The data are presented as the mean ± standard deviation from at least 3 independent experiments and were analyzed using Student’s t-tests. P<0.05 was considered to indicate a statistically significant difference.

## Results

### PG inhibited TPA-induced COX-2 in THP-1 cells

**Figure 1.**
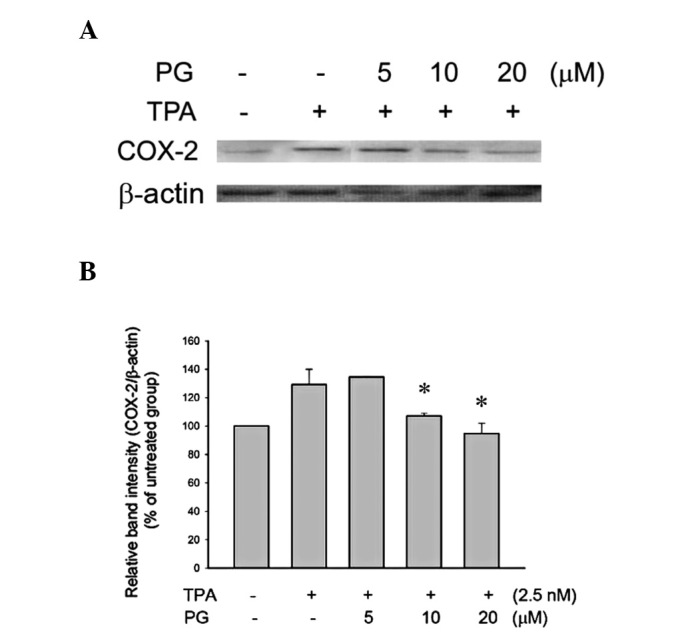
Inhibitory effects of PG on TPA-induced COX-2 protein levels in THP-1 cells. (A) The cells were treated with PG (5–20 *μ*M) for 2 h prior to treatment with 2.5 nM TPA for 24 h. (A) COX-2 expression was evaluated by western blotting; (B) COX-2 expression levels were normalized to β-actin using a densitometer. ^*^P<0.05 represents a significant difference from the cells treated with TPA only; (n=3). PG, propyl gallate; TPA, 12-O-tetradecanoylphorbol-13-acetate; COX-2, cyclooxygenase-2.

TPA is an inflammatory agent that targets intracellular protein kinase C (PKC) and induces inflammation via the activation of the NF-κB pathway. In order to evaluate the anti-inflammatory activity of PG, TPA was used to induce inflammation in THP-1 cells. Treatment with 2.5 nM TPA for 24 h increased COX-2 expression levels in the THP-1 cells. Cells that were pretreated with PG (10 or 20 *μ*M) for 2 h before the TPA treatment showed significantly inhibited COX-2 expression (Fig. 1).

### PG inhibited TPA-induced PGE2 in THP-1 cells

**Figure 2.**
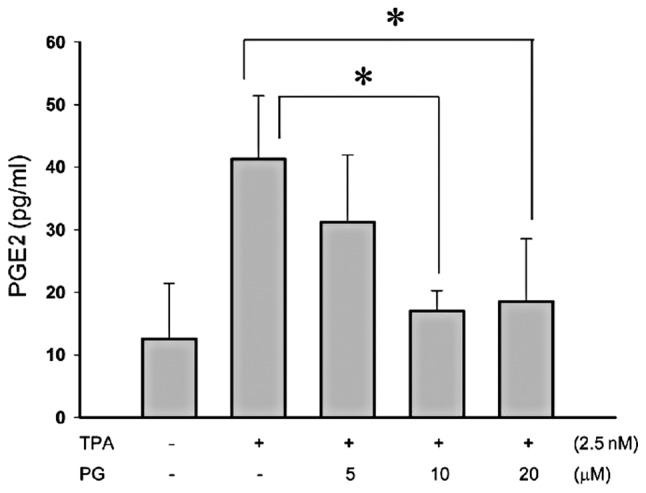
Effect of PG on TPA-induced prostaglandin PGE2 release from THP-1 cells. The cells were pretreated with PG for 2 h, then stimulated by 2.5 nM TPA for 48 h. ^*^P<0.05 represents a significant difference from the cells treated with TPA only. PG, propyl gallate; PGE2, prostaglandin E2; TPA, 12-O-tetradecanoylphorbol-13-acetate.

PGE2 is an extensively studied prostaglandin owing to its predominance in inflammation. PGE2 has been of great interest as a therapeutic target, for example, through the modulation of its synthesis by COX inhibitors. To evaluate whether PG suppresses PGE2 production in human monocytes treated with inflammatory agents, human THP-1 monocyte cell lines were pretreated with PG (5–20 *μ*M) for 2 h and then exposed to 2.5 nM TPA for 48 h. Using a PGE2 ELISA kit, it was demonstrated that pretreatment with 10 or 20 *μ*M PG significantly inhibited PGE2 production (Fig. 2). These results suggest that PG has anti-inflammatory activity.

### PG inhibited TPA-induced activation of NF-κB and nuclear translocation in THP-1 cells

**Figure 3.**
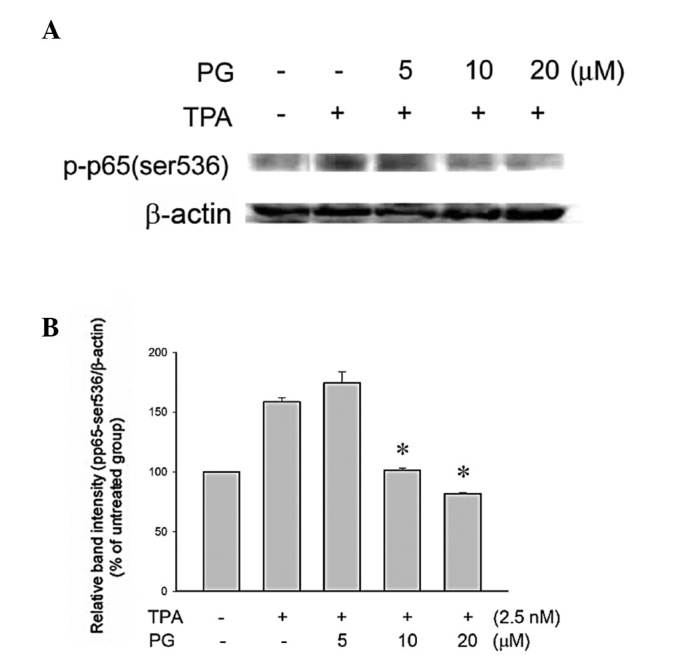
Inhibitory effects of PG on TPA-induced phosphorylation of p65 (Ser536) in THP-1 cells. The cells were pretreated with PG (5, 10 or 20 *μ*M) for 2 h, then incubated with 2.5 nM TPA for 2.5 h. (A) p-p65 expression was evaluated by western blotting; (B) p-p65 (Ser536) expression levels were normalized to β-actin using a densitometer. ^*^P<0.05 represents a significant difference from the cells treated with TPA only; (n=3). PG, propyl gallate; TPA, 12-O-tetradecanoylphorbol-13-acetate.

**Figure 4.**
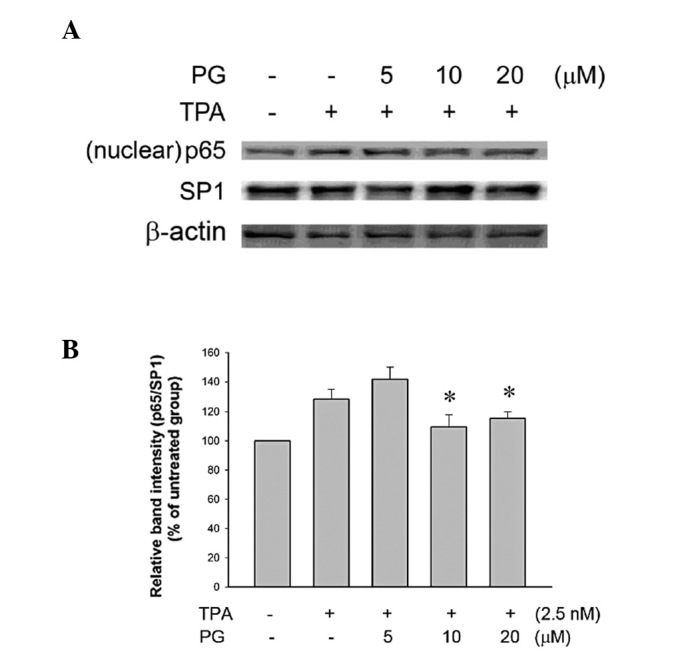
Inhibitory effects of PG on TPA-induced p65 nuclear translocation in THP-1 cells. The cells were pretreated with PG (5, 10 or 20 *μ*M) for 2 h, then incubated with 2.5 nM TPA for 4 h. (A) p65 expression in the nucleus was evaluated by western blotting; (B) p65 expression levels were normalized to β-actin using a densitometer. ^*^P<0.05 represents a significant difference from the cells treated with TPA only; (n=3). PG, propyl gallate; TPA, 12-O-tetradecanoylphorbol-13-acetate.

NF-κB, a component of the cell signaling network, is a redox-sensitive transcription factor involved in the mediation of the inflammatory response. One of the major target molecules subjected to NF-κB-driven transactivation is COX-2, which is involved in the biosynthesis of prostaglandins and inflammation (9). Therefore, whether the observed anti-inflammatory activity of PG occurred via NF-κB signaling was investigated. The transactivation of NF-κB-regulated genes requires the phosphorylation of p65/RelA (p-p65), the active subunit of NF-κB. The cells were pretreated with PG (5, 10 or 20 *μ*M) for 2 h followed by incubation with TPA (2.5 nM) for 2.5 h. The phosphorylation of p65/RelA (Ser536) was significantly inhibited by treatment with 10 or 20 *μ*M PG (Fig. 3). In addition, treatment with these two concentrations of PG (10 and 20 *μ*M) significantly inhibited the nuclear translocation of p65 (Fig. 4), indicating that the anti-inflammatory activity of PG occurred via the inhibition of the NF-κB signaling pathway.

### PG inhibited TPA-induced phosphorylation of IκB in THP-1 cells

**Figure 5.**
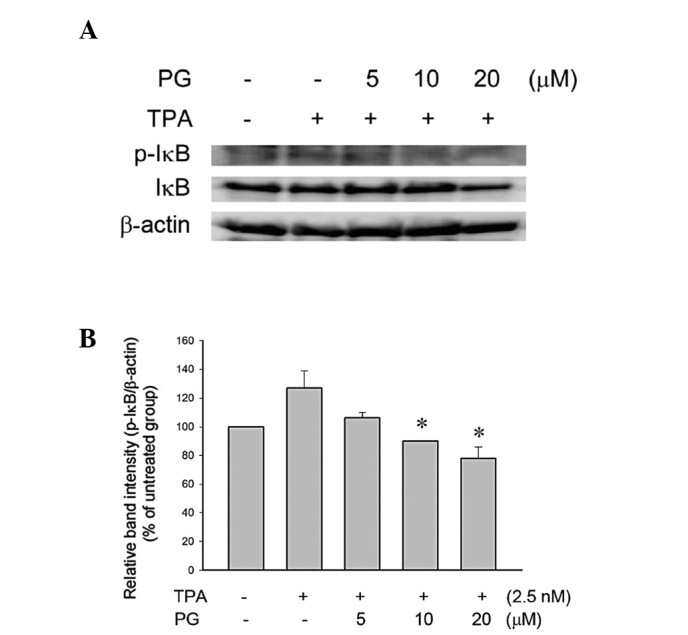
Inhibitory effects of PG on TPA-induced phosphorylation of IκB in THP-1 cells. The cells were pretreated with PG (5, 10 or 20 *μ*M) for 2 h, then incubated with 2.5 nM TPA for 2.5 h. (A) p-IκB expression was evaluated by western blotting; (B) p-IκB expression was normalized to β-actin using a densitometer. ^*^P<0.05 represents a significant difference from the cells treated with TPA alone; (n=3). PG, propyl gallate; TPA, 12-O-tetradecanoylphorbol-13-acetate.

NF-κB is bound in an inactive state in the cytoplasm by the inhibitor protein IκB (14). Following cellular stimulation by a variety of agents, IκB is phosphorylated and then degraded by the proteasome, allowing NF-κB to translocate to the nucleus and regulate the expression of the target genes. The phosphorylation of IκB was increased by the TPA treatment (Fig. 5), indicating that TPA activates NF-κB signaling via the phosphorylation of IκB. The phosphorylation of IκB was significantly inhibited by treatment with 10 or 20 *μ*M PG (Fig. 5).

### PG inhibited TPA-induced phosphorylation of IκB kinase (IKK) in THP-1 cells

**Figure 6.**
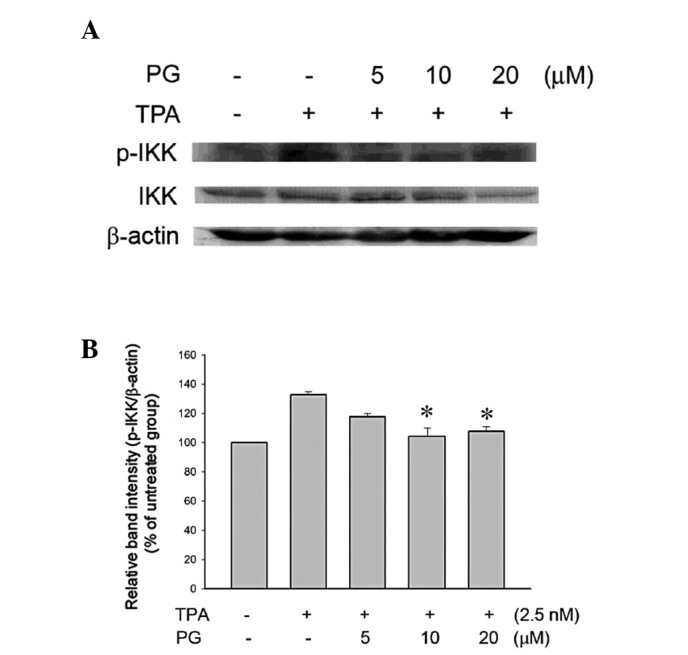
Inhibitory effects of PG on TPA-induced activation of p-IKK protein expression in THP-1 cells. The cells were pretreated with PG (5, 10 or 20 *μ*M) for 2 h, then incubated with 2.5 nM TPA for 30 min. (A) p-IKK expression was evaluated by western blotting; (B) p-IKK expression levels were normalized to β-actin using a densitometer. ^*^P<0.05 represents a significant difference from the group treated with TPA only; (n=3). PG, propyl gallate; TPA, 12-O-tetradecanoylphorbol-13-acetate; IKK, IκB kinase.

IKK is able to phosphorylate IκB and cause its degradation by ubiquitination. It was further evaluated whether PG was able to inhibit the IKK activity in TPA-treated THP-1 cells. As shown in Fig. 6, the PG treatment (10 or 20 *μ*M) significantly inhibited the phosphorylation of IKK. The expression of IKK was also inhibited by treatment with 20 *μ*M PG. These results indicated that PG inhibits the upregulated NF-κB signaling pathway, including IKK and IκB.

## Discussion

In anti-inflammatory studies, three general categories of inflammatory inducers are considered including, i) gram-negative bacterial cell wall lipopolysaccharide (LPS); ii) proinflammatory cytokines such as tumor necrosis factors (TNF-α); and iii) the tumor promoter agent TPA. With LPS, the cells are able to simulate pathogenic infections caused by inflammation. TNF-α directly binds to specific receptors (TNFR1 and TNFR2), which then activate the NF-κB signaling pathway to induce inflammation. TPA penetrates intracellularly and activates PKC and thus, the NF-κB signaling pathway is activated to induce inflammation. Compared with LPS, TPA is chemically more stable, less variable and requires a lower concentration to induce inflammation. Furthermore, unlike TNF-α, TPA does not induce caspase activation. Therefore, in the present study, TPA was selected as the inflammation inducer.

Numerous antioxidants extracted from food show excellent anti-inflammatory effects. For example, piperine, a major component of black (*Piper nigrum* Linn) and long (*Piper longum* Linn) pepper, exhibits antioxidant activity. The dose-dependent decrease of phorbol 12-myristate 13-acetate (PMA)-induced COX-2 expression and PGE2 production in murine RAW 264.7 macrophages by piperine was identified to be partially due to the inhibition of PMA-induced NF-κB nuclear translocation (18). The dietary flavonoid quercetin is an antioxidant that possesses anti-inflammatory properties. Quercetin protects cells against TNF-α-induced activation of the NF-κB signaling pathway (19). This inhibitory effect of quercetin was mediated, at least in part, by extracellular regulated kinase, c-jun amino-terminal kinase and reactive oxygen species and accompanied by reduced COX-2 levels (19).

It is notable that tannin and phenolic compounds extracted from tomatoes express excellent inhibition effects in TPA-induced COX-2 expression in KB cells (20). These results are in agreement with those of the present study showing that the anti-inflammatory effect of PG occurs via the NF-κB signaling pathway and COX-2 inhibition in TPA-treated THP-1 cells. PG belongs to the polyphenolic compound family and is synthesized by the condensation of propanol and gallic acid. In general, PG has been proposed to act as an antioxidant that protects foods against oxidation by hydrogen peroxide and oxygen free radicals. It is used in foods, cosmetics, hair products, adhesives and lubricants. Our present study shows that apart from its antioxidant activity, PG also demonstrates anti-inflammatory activity.

A number of studies have reported that gallic acid (3,4,5-trihydroxybenzoic acid), a natural polyphenol obtained from gallnuts and green tea, has antioxidant, anti-inflammatory and radical-scavenging activities. In human mast cells, gallic acid decreased the PMA plus calcium ionophore A23187-stimulated gene expression and production of proinflammatory cytokines such as TNF-α and inteleukin (IL)-6. The inhibitory effect of gallic acid on the proinflammatory cytokines was demonstrated to be dependent on NF-κB and p38 mitogen-activated protein kinase (21). Our previous study demonstrated that the antioxidant activity of PG is higher than that of gallic acid in THP-1 cells treated with various reactive oxygen species (22). Another study has demonstrated that certain derivatives of gallic acid exhibit good anti-inflammatory activity, as determined by the carrageenan-induced paw edema test (23). These studies may provide an explanation for the present results, wherein PG inhibits TPA-induced inflammatory reactions in THP-1 cells by blocking COX-2, PGE2 and IKK activity and NF-κB signaling and these effects may partially be associated with its excellent antioxidant activity.
